# PTSD Symptom Severities, Interpersonal Traumas, and Benzodiazepines Are Associated with Substance-Related Problems in Trauma Patients

**DOI:** 10.3390/jcm5080070

**Published:** 2016-08-10

**Authors:** Jeffrey Guina, Ramzi W. Nahhas, Adam J. Goldberg, Seth Farnsworth

**Affiliations:** 1 Wright-Patterson Medical Center, Wright-Patterson Air Force Base, OH 45433, USA; 2 Wright State University Boonshoft School of Medicine, Dayton, OH 45469, USA; ramzi.nahhas@wright.edu (R.W.N.); goldberg.6@wright.edu (A.J.G.); 3 Misawa Air Base Medical Center, Misawa Air Base, Japan; seth.farnsworth@gmail.com

**Keywords:** PTSD, trauma, stress disorder, substance use, alcohol, tobacco, nicotine, drugs

## Abstract

**Background:** Trauma is commonly associated with substance-related problems, yet associations between specific substances and specific posttraumatic stress disorder symptoms (PTSSs) are understudied. We hypothesized that substance-related problems are associated with PTSS severities, interpersonal traumas, and benzodiazepine prescriptions. **Methods:** Using a cross-sectional survey methodology in a consecutive sample of adult outpatients with trauma histories (*n* = 472), we used logistic regression to examine substance-related problems in general (primary, confirmatory analysis), as well as alcohol, tobacco, and illicit drug problems specifically (secondary, exploratory analyses) in relation to demographics, trauma type, PTSSs, and benzodiazepine prescriptions. **Results:** After adjusting for multiple testing, several factors were significantly associated with substance-related problems, particularly benzodiazepines (AOR = 2.78; 1.99 for alcohol, 2.42 for tobacco, 8.02 for illicit drugs), DSM-5 PTSD diagnosis (AOR = 1.92; 2.38 for alcohol, 2.00 for tobacco, 2.14 for illicit drugs), most PTSSs (especially negative beliefs, recklessness, and avoidance), and interpersonal traumas (e.g., assaults and child abuse). **Conclusion:** In this clinical sample, there were consistent and strong associations between several trauma-related variables and substance-related problems, consistent with our hypotheses. We discuss possible explanations and implications of these findings, which we hope will stimulate further research, and improve screening and treatment.

## 1. Introduction

Research consistently demonstrates high substance use disorder (SUD) rates among trauma-exposed populations with or without posttraumatic stress disorder (PTSD) [1,2,3]. However, few studies analyze associations with specific PTSD symptoms (PTSSs). To the authors’ knowledge, this is the first study to examine substances and specific PTSSs using Diagnostic and Statistical Manual of Mental Disorders (DSM)-5 criteria (though a few analyze DSM-IV PTSD clusters [4,5,6,7]). Our objective is to examine a range of potential predictors of substance-related problems among trauma patients, to better identify, monitor, and treat these particularly treatment-resistant patients. While a wide variety of substances (e.g., alcohol, tobacco, cocaine, opioids, and cannabis) have been identified as being commonly abused in patients with PTSD, and SUD of any type has been associated with worse PTSSs [8], little research has examined specific substance types and PTSSs. Previous studies found SUDs to correlate with re-experiencing [4], avoidance/numbing and arousal [5], and all three DSM-IV clusters [6,7]. We hypothesize (H1) that all substances will be associated with worse PTSSs, but expect that alcohol will be particularly associated with avoidance, numbness, and detachment (alcohol may be another form of avoidance/numbing, replacing or supplementing one’s natural ability to suppress or escape distressing thoughts and emotions [9]); and that illicit drugs will be associated with worse recklessness and irritability (drug use may cause disinhibition, or may be the result of impulsivity [10]).

Several studies have examined how specific trauma types are associated with substances. New York City residents were found to have increased smoking following the 9/11 Attacks, and those with prior tobacco use disorder were more likely to develop PTSSs [11]. Hurricane Katrina survivors reported increased coping-motivated substance use [12] and, among first responders to the 1998 Swissair Flight 111 disaster, PTSSs correlated with coping-motivated alcohol use [13]. Combat veterans have increased SUD rates, especially among those with PTSSs [14]. The prevalence of alcohol use disorder is increased amongst adults who experienced child abuse or other childhood traumas, especially among those with PTSSs [15]. Repetitive intimate partner violence, sexual assault, and physical abuse are all associated with increased PTSD-SUD comorbidity [16]. We hypothesize (H2) that substance-related problems are particularly associated with interpersonal traumas such as child abuse, and assault.

Previous studies have noted increased rates of alcohol use disorder in those treated with benzodiazepines for PTSD and anxiety [17,18]. While benzodiazepines are generally considered contraindicated in those with SUD histories, PTSD patients with comorbid SUD are more likely to receive benzodiazepines prescriptions than those without SUD [19,20]. Unfortunately, SUD is a major risk factor for benzodiazepine prescription misuse, although benzodiazepines can result in drug reinforcement and misuse even in patients without histories of SUD in conditions of continuous benzodiazepine availability [21]. We hypothesize (H3) that patients with a history of benzodiazepine prescriptions are more likely to have substance-related problems, especially with alcohol (which is particularly concerning because it is cross-tolerant with benzodiazepines and is readily available which may increase the chance of supplementing or replacing benzodiazepines with alcohol when tolerance develops [22]) and illegal drugs (those with SUD may seek prescriptions with abuse potential, but also benzodiazepine-induced disinhibition may compound PTSD-related recklessness and increase the risk of drug use [10,18,23]).

## 2. Experimental Section

### 2.1. Participants

This study was reviewed and exempted by the Wright-Patterson Air Force Base Institutional Review Board. Participants were non-emergent outpatients at a military medical center treating active duty and retired service members, and family members. Potential participants were excluded if less than 18 years old, or in apparent physical or psychological distress. Participants were recruited from October 2014 to February 2015. During clinic hours, investigators approached consecutive incoming patients in waiting areas following registration, and informally assessed exclusion criteria by observing for distress and asking about age. Each participant was asked to complete an anonymous survey onsite, and to place completed surveys into sealed envelopes in a collection box. Survey completion implied consent, as explained on the survey cover page which described the nature and purpose of the study, and which participants were encouraged to keep.

Of the 1000 surveys distributed to eligible participants, 759 (75.9%) completed portions of the survey necessary for the analysis (i.e., gender, age, ethnicity, PTSSs, trauma, and substances). Of these, 472 (62.2%) reported a history of trauma and only these were included in the study. Of those with trauma, 159 (33.7%) reported a history of trouble with substance use, 47.7% were women, and the mean age was 39.3 years. Regarding military status, 46.2% were active duty, 21.8% were retired, 24.2% were dependent, 7.8% marked “other” or did not respond. Table 1 summarizes participant characteristics by substance problem status.

### 2.2. Measures

The survey assessed PTSSs using the PTSD Checklist for DSM-5 (PCL-5), the latest version of the PCL, and a 20-item self-report measure based on DSM-5 criteria with strong reliability, validity, and internal consistency [24]. The rest of the survey contained questions of the authors’ design, based on DSM-5 [10] and surveys previously used by Sansone et al. [25]. These inquired about demographics; the DSM-5 trauma definition; twelve different trauma types (child neglect, child physical abuse, child sexual abuse, combat, motor vehicle collision, life-threatening injury or illness, natural disaster, physical assault, terrorism, sexual assault, violent death of a loved one, witnessed violence) and a write-in section; difficulty in controlling use of, or social, occupational, legal, or medical problems due to the use of eight different substance classes (alcohol; tobacco; stimulants; hallucinogens; cannabis; opioids; sedatives; inhalants), with examples of each class listed and a write-in section (i.e., “other”); and about past or present benzodiazepine prescriptions (with a list of the generic and trade names of all benzodiazepines available in the U.S.).

Participants were considered to meet DSM-5 PTSD criteria if they reported a trauma history along with at least moderate severity (2–4 on a 0–4 scale) of the following symptoms: at least one intrusion symptom, at least one avoidance symptom, at least two mood/cognitive symptoms, and at least two hyperarousal symptoms. To be conservative, less than moderate symptoms were not considered sufficient for diagnosis.

### 2.3. Statistical Analysis

Analyses were performed using logistic regression to estimate adjusted odds ratios (AORs) adjusted for gender, race, and age. The primary (confirmatory) analyses, adjusted for multiple testing, were tests of association between having a substance-related problem and each of the following explanatory variables: gender, age, race, 12 trauma types, benzodiazepine prescriptions, DSM-5 PTSD, total PTSS severity, four PTSD cluster severities, and 20 individual PTSSs. Secondary (exploratory) analyses tested for associations between these variables and each of the alcohol-, tobacco-, and illicit drug-related problems. Adjustment for multiple testing across all 42 tests in the primary analyses were conducted via the Hommel procedure [26] in SAS PROC MULTTEST [27] at the familywise Type I error rate of 0.05. All other statistical analyses were carried out in R 3.1.2 [28]. All tests were two tailed and at the α = 0.05 level of significance.

## 3. Results

Table 2 presents the AORs measuring the associations between explanatory variables and substance problem outcomes.

### 3.1. PTSS Severity (H1)

Any substance-related problem and alcohol-related problems were associated with DSM-5 PTSD criteria, total PTSS severity, all four PTSS clusters, and all twenty individual PTSSs. Tobacco-related problems were associated with all the same variables except flashbacks. Illicit drug-related problems were associated with all the PTSS-related variables except flashbacks, psychological reactivity, physical reactivity, external avoidance, amnesia, irritability, and hypervigilance. After adjusting for multiple testing for the primary analysis of any substance, DSM-5 PTSD, the total and PTSS cluster severities, and all but three of the individual PTSSs (flashbacks, physical reactivity, and hypervigilance) remained statistically significant.

### 3.2. Interpersonal Traumas (H2)

Physical assault and child neglect were significantly associated with problems related to any substance, alcohol, tobacco, and illicit drugs. Child physical abuse was significantly associated with problems related to any substance, alcohol, and tobacco. Child sexual abuse was significantly associated with problems related to any substance and tobacco. Sexual assault was significantly associated with problems related to tobacco. After adjusting for multiple testing for the primary analysis of any substance, physical assault and child physical abuse remained statistically significant.

### 3.3. Benzodiazepine Prescriptions (H3)

Benzodiazepine prescriptions were significantly associated with problems related to any substance, alcohol, tobacco, and illicit drugs. After adjusting for multiple testing for the primary analysis of any substance, the association with benzodiazepine prescriptions remained statistically significant.

## 4. Discussion

### 4.1. Worse PTSS Severity Predicts Substance-Related Problems (H1)

PTSSs—greater total and cluster severities and almost every individual PTSS—were associated with significantly greater problems with any substance (especially recklessness and negative beliefs). Also, our secondary, exploratory analyses consistently suggest that PTSSs may also be associated with problems with alcohol (especially internal avoidance and excessive startle), tobacco (especially recklessness and negative beliefs), and illicit drugs (especially negative beliefs, insomnia, and recklessness). As predicted, recklessness was associated with many substance-related problems, and alcohol was particularly associated with avoidance. Little research has focused on specific PTSSs and substance classes, but many clinicians and researchers have hypothesized that sedating substances may serve as a form of avoidance from trauma-related cognitions, emotions, and physiological reactions [29,30]. Regarding recklessness, it may be both a cause and effect of substance use. That is, substance use is an example of reckless behavior and may also contribute to further reckless behavior (e.g., disinhibition, impaired judgment, drug-related crimes). Our findings linking alcohol and excessive startle are consistent with previous research correlating arousal cluster symptoms to alcohol more so than to other substances [5], which may support the self-medication hypothesis [6] or be explained by alcohol withdrawal. Our findings linking insomnia and illicit drugs are consistent with previous research and may have a bidirectional causation (e.g., using substances to self-medicate insomnia, and substances impairing sleep) [5]. Finally, one of the most strongly associated symptoms across all substance categories was negative beliefs. Potential explanations for this finding include those with a sense of a foreshortened future (a DSM-IV PTSD symptom subsumed by negative beliefs in DSM-5) being less deterred from use by prospects of the long-term negative impacts of substance use. Different substances may be used by different individuals for different reasons (e.g., self-medicating certain symptoms), although self-medication is certainly not the only explanation considering that patients frequently use drugs like cocaine which are associated with a worsening of PTSD symptoms [31] and which may support the reward deficiency hypothesis [32]. Further research is needed to better elucidate the potential explanations for our findings. We also hope that future research will examine if screening for these symptoms will improve the detection of undetected substance problems in trauma patients and undetected trauma histories in substance using patients, and if treatments targeting these symptoms will improve outcomes.

### 4.2. Interpersonal Traumas Predict Substance-Related Problems (H2)

As expected, we found assaults and child abuse to be significantly associated with substance-related problems. Across both the primary and secondary analyses, we found associations between physical assault and child neglect with all substance categories, child abuse (physical or sexual) with most substances, and sexual traumas (child abuse or assault) with tobacco. Developmental traumas have previously been associated with substance use in general [33], and childhood sexual abuse with tobacco specifically [34], but most of these findings are novel. Early development is important in determining one’s response to stressors later in life, and may help explain the increased vulnerability for substance problems in survivors of childhood trauma [35]. Child abuse has been associated with depression, fear, and hopelessness, which disrupt positive coping skill development and encourage maladaptive coping skills such as substance use [36]. In addition to psychological factors, biological factors (e.g., genetic, endocrinologic, immunologic) have been linked to the physical impact of childhood trauma on the developing brain [37], which could potentially predispose survivors to substance problems, though further research is needed to determine if these factors help explain our findings, or have utility in clinical practice.

### 4.3. Benzodiazepine Prescriptions Predict Substance-Related Problems (H3)

As we hypothesized, current or past benzodiazepine prescriptions had among the strongest associations with substance-related problems. When including our exploratory analyses, this association was especially strong with illicit drugs (AOR = 8.02). Again, the correlation may involve bidirectional causation: prescriptions could cause iatrogenic addiction or disinhibition resulting in substance problems; or those with substance-related problems may seek out prescriptions. Regarding the former hypothesis, iatrogenic dependence occurs in 58%–100% of those prescribed long-term therapeutic doses, including many without any history of SUDs [10,38,39]. As tolerance develops, some patients are able to persuade prescribers to increase their dose, but when tolerance or withdrawal cannot be assuaged through prescriptions (from one prescriber or by “doctor shopping”), some obtain benzodiazepines through the internet, the black market (“the street”), theft (e.g., from manufacturers, delivery services, medical facilities, pharmacies, or patients’ medicine cabinets), or supplement use with alcohol or other substances [10,38,40]. This progression may be more common in PTSD patients, who often seek avoidance of negative thoughts/feelings (self-medication hypothesis [6]) or reckless reward through substances (reward deficiency hypothesis [32]). Our findings are consistent with previous studies demonstrating benzodiazepine use in trauma patients is associated with worse outcomes, including substance use [29]. Prescribing benzodiazepines in trauma-exposed populations is complicated by high rates of comorbid SUDs and, if treatment is given without addressing underlying substance use (or substance-induced symptoms such as anxiety), further addictive behaviors can develop [41]. While these findings are preliminary, taken together with past findings and recommendations that benzodiazepines are relatively contraindicated in trauma patients [29] and with risks of legal liability [42], benzodiazepines should be avoided or at least used with caution in patients with PTSD or trauma histories.

### 4.4. Substance-Related Problems More Common in Traumatized Men

Among those with trauma, men were more likely than women to have problems with at least one substance, especially alcohol (although in the primary analysis this association was not statistically significant after adjusting for multiple testing). This is consistent with previous research demonstrating that, within trauma-exposed populations, men are more likely to develop externalizing symptoms (e.g., impulsivity, substance use) while women are more likely to internalize (e.g., anxiety, depression) [43,44,45]. Our findings may also potentially be explained by evidence that alcohol has greater anxiety-reducing effects on men than women [44], though further research is necessary. Surprisingly, we observed the opposite relationship for illicit drugs—for which women had more problems—though the difference between genders was not significantly different.

### 4.5. Limitations

Limitations of our study include a cross-sectional approach, self-report reliability, use of investigator-designed questionnaires (other than the PCL-5), and generalizability which render these findings preliminary. We surveyed a clinical military sample, which may explain the small sample size of those reporting illicit drugs as strict drug policies in the military may deter use or reporting use—although we tried to improve self-report reliability with anonymous surveys (participants were assured confidentiality on the survey cover page). There are many possible interaction terms which could impact results and, while adjusting for age, gender, and race can reduce confounding, it could be that any of these factors are effect modifiers; this could be thoroughly examined by future research. While the primary analyses were adjusted for the multiplicity of testing, the analyses of alcohol-, tobacco-, and illicit-drug-related problems were exploratory. The primary analyses may be considered confirmatory with the caveats that this was an observational study, and therefore subject to potential confounding from unobserved variables, and cross-sectional, and therefore the direction of causality cannot be established. Because there is a paucity of studies examining specific substances and PTSSs, we believe this current brief report adds to the existing literature with its large sample size and by exploring these associations in relation to the DSM-5 PTSD criteria (which also allowed for avoidance and numbing to be analyzed separately which researchers have long called for to better understand substance-related associations [31]). Furthermore, we believe our findings warrant further investigation for confirmation (in particular, of our exploratory analyses), determination of correlation vs. causation, generalizability to other populations, use of more validated measures (e.g., for trauma type), and to improve patient screening and clinical care (e.g., targeting certain symptoms to diagnose, prevent, and treat substance problems in trauma patients).

## 5. Conclusions

In this clinical sample of trauma patients, we found high levels of substance-related problems which were significantly associated with most PTSSs (particularly negative beliefs, recklessness, and avoidance), interpersonal trauma types (assaults and child abuse), and benzodiazepine prescriptions. Considering the mortality, morbidity, and societal costs of trauma and substance problems, it is important that future research investigates these links to improve screening and treatment targets.

## Figures and Tables

**Table 1:** Participant characteristics, by history of substance-related problems.

	Substance Problems	No Substance Problems
Participants, *n*	159	313
Male, *n* (%)	96 (60.4)	151 (48.2)
Age, years, mean (sd)	40.3 (13.9)	38.8 (13.3)
**Race, *n* (%)**		
White	122 (76.7)	249 (79.6)
Black	18 (11.3)	39 (12.5)
Hispanic	9 (5.7)	8 (2.6)
Asian	1 (0.6)	5 (1.6)
Native American	1 (0.6)	1 (0.3)
Other	1 (0.6)	4 (1.3)
Multiracial	7 (4.4)	7 (2.2)
**Military status, *n* (%)**		
Active duty	65 (40.9)	153 (48.9)
Retired	40 (25.2)	63 (20.1)
Dependent	37 (23.3)	77 (24.6)
Other/Not specified	17 (10.7)	20 (6.4)
**Trauma type, *n* (%)**		
Witnessed violence	67 (42.1)	107 (34.2)
Life-threatening injury/illness	67 (42.1)	95 (30.4)
Combat	51 (32.1)	91 (29.1)
Physical assault	55 (34.6)	57 (18.2)
Sexual assault	36 (22.6)	71 (22.7)
Child sexual abuse	36 (22.6)	49 (15.7)
Motor vehicle collision	33 (20.8)	52 (16.6)
Child physical abuse	39 (24.5)	44 (14.1)
Natural disaster	33 (20.8)	33 (10.5)
Child neglect	29 (18.2)	32 (10.2)
Violent death of loved one	23 (14.5)	38 (12.1)
Terrorism	25 (15.7)	32 (10.2)
Prescribed benzodiazepines, *n* (%)	60 (37.7)	66 (21.2)
**Substance problems, *n* (%)**		
Alcohol	104 (65.4)	0.0 (0.0)
Tobacco	96 (60.4)	0.0 (0.0)
Illicit drugs	31 (19.5)	0.0 (0.0)
Opioids	16 (10.1)	0.0 (0.0)
Cannabis	14 (8.8)	0.0 (0.0)
Stimulants	11 (6.9)	0.0 (0.0)
Sedatives	7 (4.4)	0.0 (0.0)
Hallucinogens	4 (2.5)	0.0 (0.0)
Inhalants	1 (0.6)	0.0 (0.0)
Other	4 (2.5)	0.0 (0.0)

**Table 2:** Adjusted odds ratios for associations between explanatory variables and substance-related problems (adjusted for gender, race, and age).

Risk Factor	Any Substance	Alcohol	Tobacco	Illicit Drugs
Men (vs. Women)	1.66 *	1.89 **	1.51	0.65
White (vs. Non-white)	0.77	0.62	0.99	0.82
+10 year age difference	1.09	0.98	1.02	0.94
**Trauma type**				
Witnessed violence	1.36	0.98	1.39	2.01
Life-threatening injury/illness	1.64 *	1.63 *	1.39	3.30 **
Combat	0.86	0.82	0.98	1.65
Physical assault	2.71 ***^,†^	2.18 **	2.47 ***	2.34 *
Sexual assault	1.40	1.22	1.98 *	0.93
Child sexual abuse	1.92 *	1.03	2.77 ***	2.21
Motor vehicle collision	1.19	0.90	1.05	1.26
Child physical abuse	2.27 **^,†^	1.81 *	2.75 ***	1.58
Natural disaster	2.01 *	1.74	1.48	0.73
Child neglect	2.02 *	2.13 *	1.99 *	3.63 **
Violent death of loved one	1.20	1.15	0.96	1.65
Terrorism	1.42	1.53	1.61	0.93
Benzodiazepine prescriptions	2.78 ***^,†^	1.99 **	2.42 ***	8.02 ***
DSM-5 PTSD	1.92 **^,†^	2.38 ***	2.00 **	2.14 *
Total PTSS severity	1.03 ***^,†^	1.03 ***	1.03 ***	1.03 **
**PTSS cluster severity**				
Intrusion	1.09 ***^,†^	1.09 ***	1.09 ***	1.08 *
Avoidance	1.20 ***^,†^	1.22 ***	1.20 ***	1.16 *
Mood/cognitive	1.07 ***^,†^	1.07 ***	1.07 ***	1.08 ***
Arousal	1.08 ***^,†^	1.08 ***	1.08 ***	1.08 **
**PTSS severity**				
Intrusive memories	2.36 ***^,†^	2.26 ***	2.77 ***	2.11 *
Nightmares	2.46 ***^,†^	2.19 ***	2.53 ***	2.39 *
Flashbacks	1.89 **	2.26 **	1.65	1.17
Psychological reactivity	2.13 ***^,†^	2.24 ***	2.58 ***	1.69
Physical reactivity	1.71 **	1.62 *	1.96 **	1.78
Internal avoidance	2.36 ***^,†^	2.87 ***	2.24 ***	2.14 *
External avoidance	2.19 ***^,†^	2.21 ***	2.40 ***	1.77
Amnesia	2.26 ***^,†^	2.79 ***	1.78 *	1.46
Negative beliefs	2.70 ***^,†^	2.42 ***	2.82 ***	3.79 ***
Blame	2.49 ***^,†^	2.52 ***	1.98 **	2.67 **
Negative feelings	2.39 ***^,†^	2.55 ***	2.78 ***	3.17 **
Anhedonia	1.98 ***^,†^	1.72 *	2.20 ***	2.36 *
Detachment	2.02 ***^,†^	1.89 **	1.99 **	2.81 **
Emotional numbness	2.23 ***^,†^	2.02 **	2.44 ***	2.44 *
Irritability/aggression	2.16 ***^,†^	2.58 ***	2.16 **	1.67
Recklessness	2.85 ***^,†^	2.79 ***	2.86 ***	3.38 **
Hypervigilance	1.72 **	2.01 **	2.08 **	1.21
Excessive startle	2.33 ***^,†^	2.87 ***	2.35 ***	2.34 *
Inattention	2.11 ***^,†^	2.13 ***	2.37 ***	3.08 **
Insomnia	1.87 **^,†^	2.14 **	1.91 **	3.69 **

* *p* < 0.05; ** *p* < 0.01; *** *p* < 0.001; ^†^ Significantly different (at the familywise Type I error rate of 0.05) after adjusting for multiple comparisons (for the primary “Any Substances” analysis).

## Abbreviations

The following abbreviations are used in this manuscript:

AOR	Adjusted Odds Ratio
PTSD	Post-traumatic Stress Disorder
PTSS	Post-traumatic Stress Symptom
SUD	Substance Use Disorder
